# A Nanobody-Mediated Virus-Targeting Drug Delivery Platform for the Central Nervous System Viral Disease Therapy

**DOI:** 10.1128/Spectrum.01487-21

**Published:** 2021-11-24

**Authors:** Song Zhu, Fei Luo, Bin Zhu, Fei Ling, Er-Long Wang, Tian-Qiang Liu, Gao-Xue Wang

**Affiliations:** a College of Animal Science and Technology, Northwest A&F University, Yangling, China; b College of Fisheries, Southwest University, Chongqing, China; c Key Laboratory of Freshwater Fish Reproduction and Development (Ministry of Education), College of Life Sciences, Southwest University, Chongqing, China; d Key Laboratory of Aquatic Science of Chongqing, College of Life Sciences, Southwest University, Chongqing, China; Regional Centre for Biotechnology

**Keywords:** nanobody, carbon nanotube, viral encephalopathy and retinopathy, central nervous system viral diseases, targeted therapy

## Abstract

Viral diseases of the central nervous system (CNS) represent a major global health concern. Difficulties in treating these diseases are caused mainly by the biological tissues and barriers, which hinder the transport of drugs into the CNS. To counter this, a nanobody-mediated virus-targeting drug delivery platform (SWCNTs-P-A-Nb) is constructed for CNS viral disease therapy. Viral encephalopathy and retinopathy (VER), caused by nervous necrosis virus (NNV), is employed as a disease model. SWCNTs-P-A-Nb is successfully constructed by employing single-walled carbon nanotubes, amantadine, and NNV-specific nanobody (NNV-Nb) as the nanocarrier, anti-NNV drug, and targeting ligand, respectively. Results showed that SWCNTs-P-A-Nb has a good NNV-targeting ability *in vitro* and *in vivo*, improving the specific distribution of amantadine in NNV-infected sites under the guidance of NNV-Nb. SWCNTs-P-F-A-Nb can pass through the muscle and gill and be excreted by the kidney. SWCNTs-P-A-Nb can transport amantadine in a fast manner and prolong the action time, improving the anti-NNV activity of amantadine. Results so far have indicated that the nanobody-mediated NNV-targeting drug delivery platform is an effective method for VER therapy, providing new ideas and technologies for control of the CNS viral diseases.

**IMPORTANCE** CNS viral diseases have resulted in many deadly epidemics throughout history and continue to pose one of the greatest threats to public health. Drug therapy remains challenging due to the complex structure and relative impermeability of the biological tissues and barriers. Therefore, development in the intelligent drug delivery platform is highly desired for CNS viral disease therapy. In the study, a nanobody-mediated virus-targeting drug delivery platform is constructed to explore the potential application of targeted therapy in CNS viral diseases. Our findings hold great promise for the application of targeted drug delivery in CNS viral disease therapy.

## INTRODUCTION

Although the central nervous system (CNS) is well protected by biological tissues and barriers, a wide variety of viruses have developed diverse strategies to enter the CNS, such as poliovirus, rabies virus, and influenza virus (1–3). In addition, emerging viruses are continually increasing and challenging the CNS in new ways (1). For example, SARS-CoV-2, the causative agent of coronavirus disease 2019, infects not only the respiratory system but also the nervous system (4). Actually, the number of CNS viral diseases each year is greater than that of all bacterial, fungal, and protozoa infections combined (5). This shows that the CNS viral diseases have become a major global public health concern, and effective measures are urgently needed.

At present, the therapy of CNS viral diseases is still a worldwide problem. Generally, drug therapy is an effective measure for viral diseases control, while it remains challenging for CNS viral diseases (6). One of the biggest challenges is caused by the biological tissues and barriers, which hinder the transport of drugs into the CNS (7, 8), such as the blood-brain barrier (BBB). Therefore, development in the intelligent drug delivery platform is highly desired for the CNS viral diseases therapy.

Nano-targeted drug delivery system refers to the use of nanocarriers to transport drugs to the specific sites under the guidance of targeting ligands (9, 10). As one of the most promising nanocarriers, carbon nanotubes (CNTs) have been used widely in drug delivery attributable to their excellent properties, such as high carrying capacity, needle-like structure, and strong penetrability (11, 12). CNTs have a higher intercellular diffusion rate than globular nanoparticles with similar weight (13). Moreover, CNTs are uniquely equipped to carry drugs across biological tissues and barriers, including the BBB (14, 15), and have been extensively used in targeting therapy for CNS diseases (16, 17). For targeted drug delivery, nanocarriers need to conjugate with targeting ligands, which can specifically recognize and bind to the targets. Antibody is one of the most popular targeting ligands (18, 19). However, the application of traditional antibody as targeting ligand is restricted by factors such as large molecular weight, strong immunogenicity, and high production cost. Fortunately, the inherent characteristics of nanobody (Nb) just make up for the deficiencies of traditional antibodies, including small size (approximately 1/10 of traditional antibodies), weak immunogenicity, improved robustness, and ease of expression in various hosts (20–22), making it an ideal targeting ligand. Presently, the most mature and widely explored area for targeted drug delivery lies in cancer therapy, while applications against CNS viral diseases are rarely reported (23).

In the study, viral encephalopathy and retinopathy (VER), caused by nervous necrosis virus (NNV), is selected as a disease model to explore the potential of targeted drug delivery in CNS viral disease therapy. Our previous work showed that amantadine has strong anti-NNV activity both *in vitro* and *in vivo*. In addition, a specific Nb (namely, NNV-Nb) without neutralizing activity against NNV was obtained from an immunized phage-displayed Nb library (unpublished results). Based on these factors, single-walled carbon nanotubes (SWCNTs), amantadine, and NNV-Nb were, respectively, employed as the nanocarrier, anti-NNV drug, and targeting ligand to construct a targeted drug delivery platform. NNV-targeting and anti-NNV activity of the platform were evaluated systemically *in vitro* and *in vivo*. The study will provide new ideas and technologies for the CNS viral disease therapy.

## RESULTS AND DISCUSSION

In the study, the potential application of targeted drug delivery technology in CNS viral diseases therapy was explored employing VER as a disease model attributed to the following. (i) NNV is a neurotropic virus, and viral replication is restricted almost entirely to nerve tissues, preferentially brain (24, 25). (ii) The main host of NNV is a variety of fishes. Numerous studies have proven fish to be a good vertebrate CNS disease model with various advantages, such as high reproduction capability, low cost, and highly conserved nature of both the genetics and cell biology compared with those of higher vertebrates (26, 27). (iii) NNV is one of the smallest and simplest animal viruses. NNV is a nonenveloped RNA virus with a diameter of 25 to 30 nm (28). Its gene and structure characteristics have been studied systematically (25, 29), making it a good CNS virus model.

Generally, targeted drug delivery system is composed mainly of nanocarriers, drugs, and targeting ligands. SWCNTs can enter into fish tissues and the CNS via bathing (23, 30). Our unpublished data showed that amantadine has a strong anti-NNV activity. Moreover, a specific Nb (NNV-Nb) without neutralizing activity against NNV was acquired from an immunized phage-displayed Nb library by affinity screening. A nanobody-mediated NNV-targeting drug delivery platform was constructed employing SWCNTs, amantadine, and NNV-Nb as the nanocarrier, anti-NNV drug, and targeting ligand, and its NNV-targeting and activity anti-NNV activity were evaluated systemically.

### Expression and affinity analysis of NNV-Nb.

For targeted drug delivery, nanocarriers need to conjugate with targeting ligands, which can specifically recognize and bind to the targets. As one of the most popular targeting ligands, antibody has been widely used owing to its excellent properties (18). However, the application of traditional antibody as targeting ligand is restricted by factors such as large molecular weight, strong immunogenicity, and high production cost. Hamers-Casterman et al. (22) found that a variable domain of heavy-chain antibodies (HCAbs) that are devoid of light chains exists in camelids (camels, llamas, alpacas). The antigen-binding site of HCAbs is composed of one single domain, referred to as Nb, which is considered to be the smallest antibody with complete antigen-binding function (22, 31). The characteristics of Nb have just covered the shortages of traditional antibodies, such as small molecular weight (∼15 kDa), strong tissue penetration, weak immunogenicity, good solubility, and easy expression in various hosts (20, 21), making it an ideal targeting ligand.

Specific Nb can be acquired from nanobody libraries by affinity screening. Nanobody libraries include the naive library and the immunized library. Generally, the naive library is easy to operate without immunization, but the Nb acquired from naive library has a weaker affinity to the target compared with that from immunized library. In addition, more types of Nb with strong affinity can be acquired from the immunized library (21, 32). An immunized phage-displayed nanobody library was previously constructed by immunization alpaca with purified NNV. Twenty-two phage clones with strong binding activity to NNV were acquired, and the neutralizing activity of Nbs was checked (unpublished data). In the study, a specific Nb, NNV-Nb, without neutralizing activity against NNV was selected as the targeting ligand.

**FIG 1:**
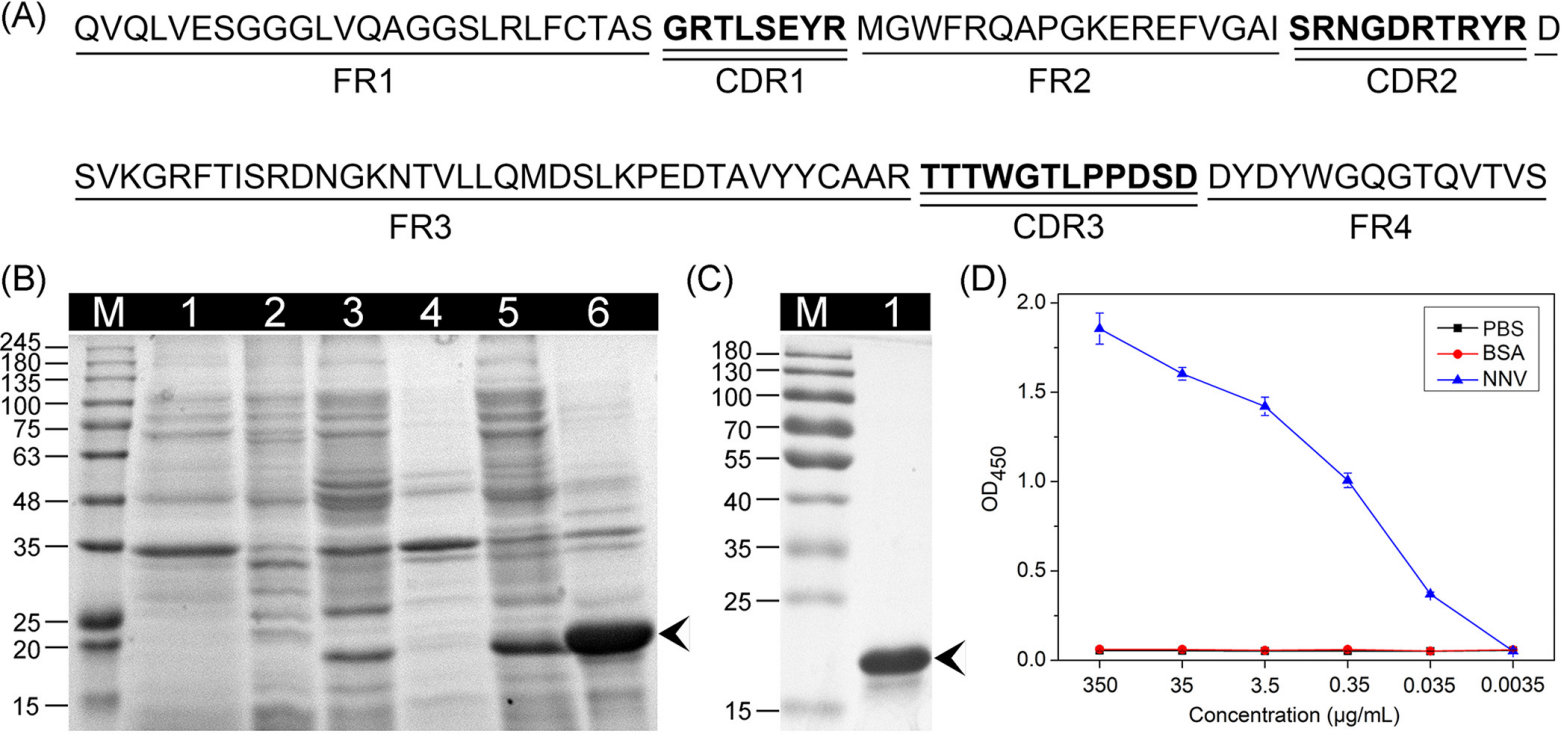
Expression and affinity analysis of NNV-Nb. (A) The deduced amino acid sequences of NNV-Nb. The framework regions (FRs) are marked by single underline, and the complementarity determining regions (CDRs) are marked by bold and double underline. (B) SDS-PAGE analysis of the expression of NNV-Nb. lane M: marker; lane 1: pET25b(+)-only supernatant; lane 2: pET25b(+)-only sediment; lane 3: pET25b-NNV-Nb supernatant without IPTG induction; lane 4: pET25b-NNV-Nb sediment without IPTG induction; lane 5: pET25b-NNV-Nb supernatant with IPTG induction; lane 6: pET25b-NNV-Nb sediment with IPTG induction. (C) SDS-PAGE analysis of the purified NNV-Nb. (D) Affinity of the purified NNV-Nb to NNV checked by indirect ELISA. Data are presented as mean ± SD. *n *= 8 per group.

The deduced amino acid sequence of NNV-Nb is shown in Figure 1A. NNV-Nb is composed of four framework regions (FRs) and three complementarity determining regions (CDRs), which is similar to the variable domain of the heavy chain (VH) of traditional antibodies. However, the CDR1 and CDR3 of Nb are larger than those of VH, not only providing a sufficiently large antigen interacting surface but also forming a variety of paratope structures to recognize special antigenic epitope (21, 33). As shown in Figure 1B, NNV-Nb can be detected in supernatant and sediment fractions of the ruptured cells after induction by IPTG (isopropyl-β-d-thiogalactopyranoside). The molecular weight of NNV-Nb is approximately 18 kDa, which is consistent with the theoretical molecular weight and another study (34). NNV-Nb in the supernatant fraction was purified using Ni-chelating affinity chromatography and analyzed by SDS-PAGE (Fig. 1C).

Affinity of the purified NNV-Nb to NNV was checked by indirect enzyme-linked immunosorbent assay (ELISA). As shown in Figure 1D, NNV-Nb exhibited a binding ability to NNV even at 0.035 μg/mL. Previously, an NNV-specific Nb was acquired from a naive library, and results showed that the Nb had no binding ability to NNV below 3.125 μg/mL (30). The data indicated that Nb acquired from naive library has an obviously weaker affinity compared with that from immunized library.

### Construction and characterization of the drug delivery system.

As one of the most promising nanocarriers, CNTs have been wildly used owing to their excellent properties, such as strong tissue penetration, high carrying capacity, and needle-like structure (15). CNTs have an intercellular diffusion rate higher than that of globular nanoparticles with similar weight due to their high aspect ratio and needle-like structure (13). CNTs are uniquely equipped to carry drugs and other ligands across biological membranes; particularly, they have shown an intrinsic ability to cross the BBB *in vitro* and *in vivo* (14). In addition, CNTs can effectively penetrate the fish epidermis and enter into various tissues (35) and the CNS (30) by immersion. Drugs, proteins, and nucleic acids can be conjugated with CNTs by covalent or noncovalent interaction (17, 18). Chemical compounds with extended π-structures can easily be bound to CNTs by π-π interactions (36), such as fluorescein isothiocyanate (FITC).

**FIG 2:**
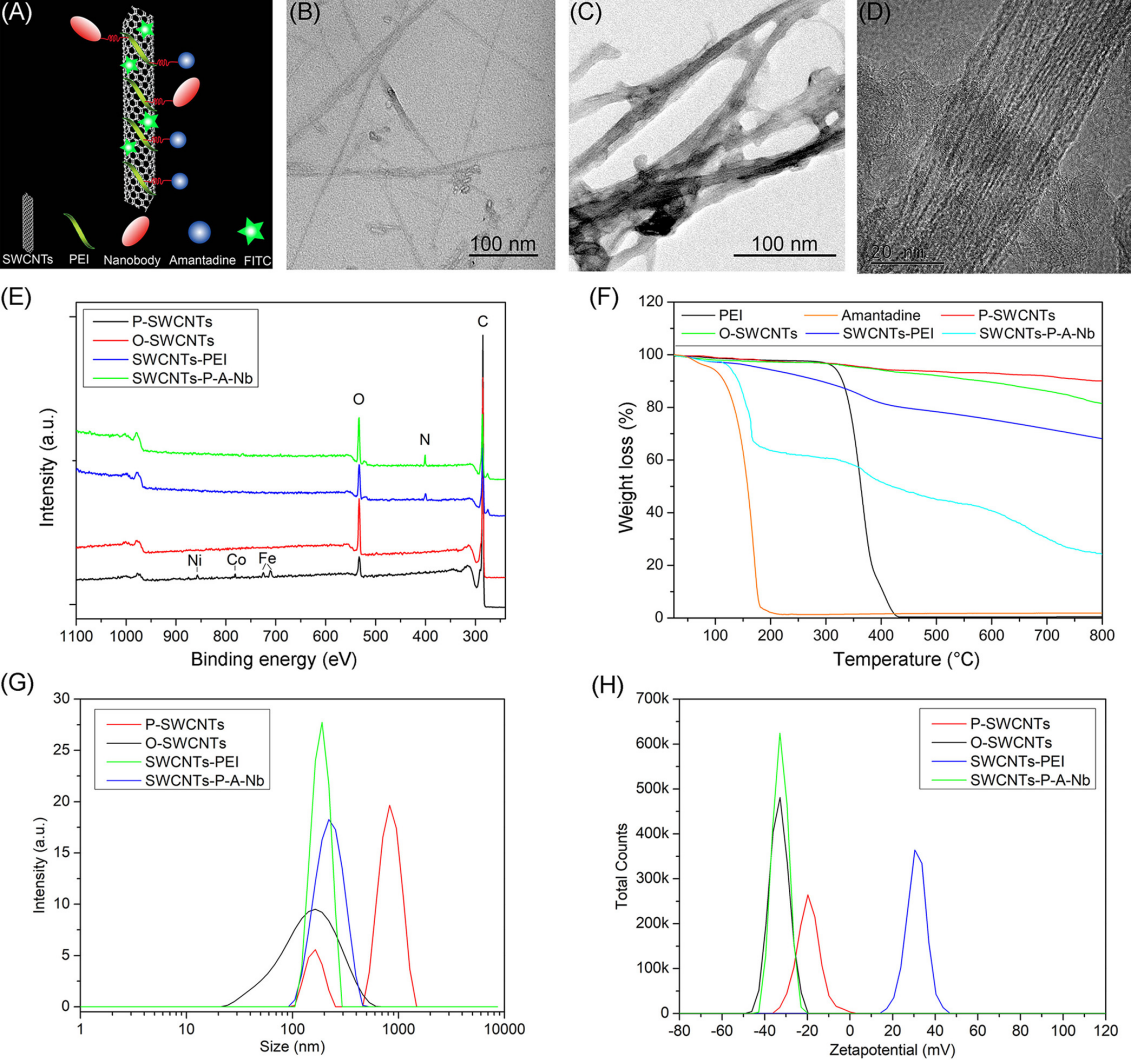
Construction and characterization of the drug delivery system. (A) Schematic presentation of the drug delivery system. Representative transmission electron microscopy images of P-SWCNTs (B) and SWCNTs-P-A-Nb (C and D). Characterization of the constructs using X-ray photoelectron spectroscopy (E), thermogravimetric analysis (F), and nano-particle size and Zeta potential analysis (G: particle size; H: zeta potential).

In the present work, SWCNTs, amantadine, and NNV-Nb were, respectively, selected as the nanocarrier, anti-NNV drug, and targeting ligand to construct a targeted drug delivery platform (Fig. 2A). To improve the dispersibility and biocompatibility, SWCNTs were first oxidized by a H_2_SO_4_/HNO_3_ mixture and then functionalized with polyethylenimine (PEI). In addition, PEI provides a large number of active amino groups for subsequent reactions (37). NNV-Nb was linked on the outermost layer using butanedioic anhydride as a linker to prevent adverse effects on the binding activity. FITC was conjugated by π-π interactions.

As shown in Figure 2B, the P-SWCNTs were fibrous with various lengths. For SWCNTs-P-A-Nb, an obvious layer around SWCNTs surface was observed visually (Fig. 2C and D), indicating that PEI, amantadine, and NNV-Nb may be conjugated on the surface of SWCNTs. To further verify the conjugation, SWCNTs-P-A-Nb was analyzed by X-ray photoelectron spectroscopy (XPS). As shown in Figure 2E, SWCNTs surface consisted mainly of carbon (C; 284 eV), as well as small amounts of oxygen (O; 532 eV), iron (Fe; 711 [Fe 2p1/2] and 725 [Fe 2p3/2] eV), cobalt (Co; 782 eV), and nickel (Ni; 850 eV). For O-SWCNTs, only carbon and oxygen can be identified from the spectrum. Surface oxygen contents for P-SWCNTs and O-SWCNTs were 3.18 and 14.10 atomic percent, indicating that the oxidation of SWCNTs was sufficient, and the impurities were removed. Nitrogen (399 eV) can be identified from the spectrum of SWCNTs-PEI, indicating that PEI was successfully conjugated with SWCNTs. Surface nitrogen contents for SWCNTs-PEI and SWCNTs-P-A-Nb were 6.81 and 7.23%, indicating that the SWCNTs-P-A-Nb was successfully constructed. The loading efficiencies of amantadine and NNV-Nb were 37.92% and 15.03%, respectively.

As shown in Figure 2F, PEI and amantadine were absolutely degraded at 430 and 220°C, respectively. P-SWCNTs showed a better thermostability than O-SWCNTs because the carboxyl groups and other functional groups of O-SWCNTs are unstable at high temperature. Based on the weight losses of O-SWCNTs (6.61%) and SWCNTs-PEI (19.71%) at 430°C, it can be calculated that the loading efficiency of PEI on SWCNTs was about 13.10%. Previously, PEI was loaded on multiwalled carbon nanotubes (MWCNTs), and the loading efficiency was 9.11% (30). The data indicate that SWCNTs have a higher PEI loading efficiency than MWCNTs, which may be attributed to the larger specific surface area of SWCNTs (36). The loading efficiencies of FITC were 8.34% and 6.41% for SWCNTs-P-F-A and SWCNTs-P-F-A-Nb, respectively.

The particle sizes and zeta potentials of the constructs were measured to check their stability. As shown in Figure 2G and Table 1, the average sizes for P-SWCNTs, O-SWCNTs, SWCNTs-PEI, and SWCNTs-P-A-Nb were 722.86, 136.97, 187.39, and 221.15 nm, respectively. The data indicate that P-SWCNTs were easily aggregated in water due to the hydration and reduction of electrostatic repulsion (38). The dispersibility was significantly improved following oxidization and PEI conjugation, which is consistent with the results reported in a previous study (39). The sizes were gradually increased following amantadine and NNV-Nb conjugation, suggesting the successful conjugation. Zeta potential analysis (Fig. 2H and Table 1) revealed a negative surface charge (−19.06 mV) for P-SWCNTs, which decreased to −49.90 mV following oxidization due to the increase of carboxyl group. After PEI conjugation, the zeta potential was increased to +33.59 mV. The zeta potential was decreased to −36.20 mV for SWCNTs-P-A-Nb due to the negative charge of NNV-Nb. The data further proof that the system was constructed successfully.

**TABLE 1:** Average particle sizes and zeta potentials of the constructs

Sample	Particle size (nm)	Zeta potential (mV)
P-SWCNTs	722.86	−19.06
O-SWCNTs	136.97	−49.90
SWCNTs-PEI	187.39	+33.59
SWCNTs-P-A-Nb	221.15	−36.20

### NNV targeting of the drug delivery system.

**FIG 3:**
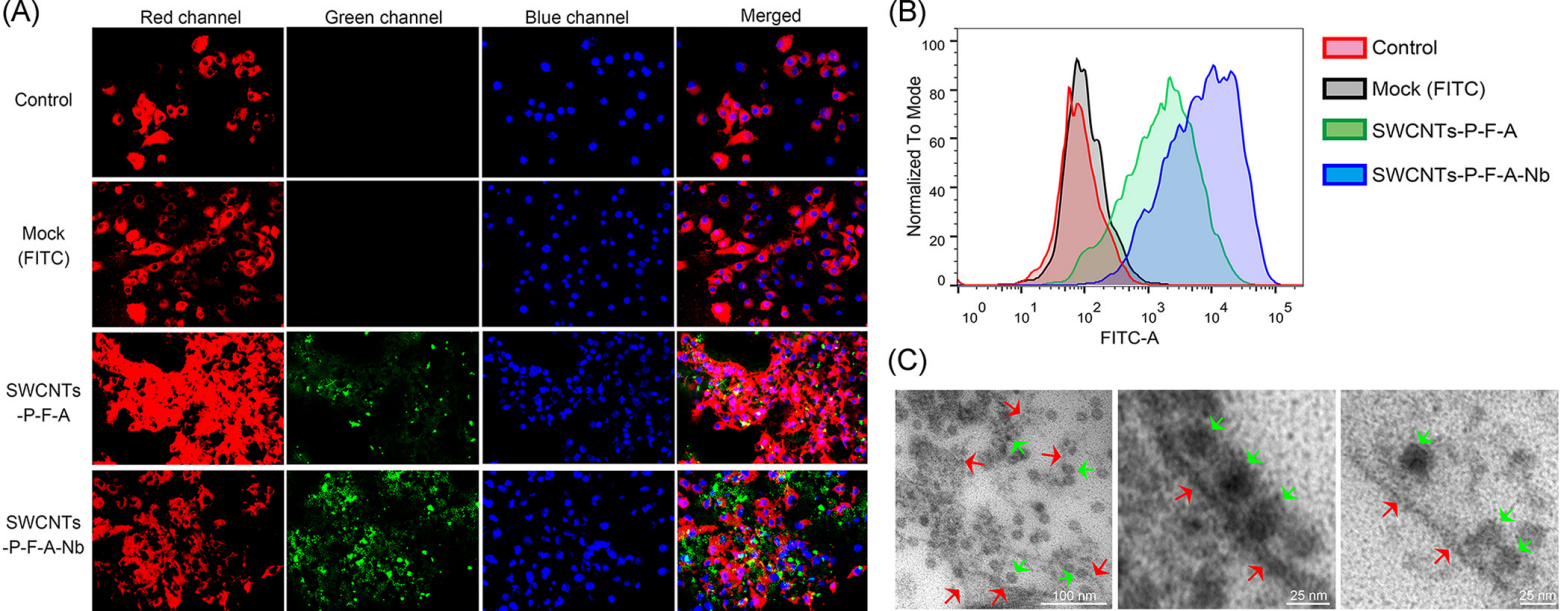
NNV targeting of the drug delivery system *in vitro*. (A) Fluorescence microscope images of the NNV-infected cells incubated with FITC (as the mock), SWCNTs-P-F-A, and SWCNTs-P-F-A-Nb with the same FITC concentration (1.0 μg/mL), cells incubated with only maintenance medium were employed as the control. Cell membrane and nucleus were, respectively, stained with Dil (red fluorescence) and DAPI (blue fluorescence), and the green fluorescence corresponds to the FITC labeling on the drug delivery systems. (B) Flow cytometry analysis of the treated SSN-1 cells without Dil and DAPI staining. (C) Specific recognition and binding ability of SWCNTs-P-A-Nb to NNV observed under a TEM. Green arrows: NNV; red arrows: SWCNTs-P-F-A-Nb.

The specific recognition and binding ability of drug delivery system to the targets is the basis and premise of targeted therapy. Therefore, the NNV targeting of SWCNTs-P-A-Nb was well checked before the anti-NNV activity evaluation. As shown in Figure 3A, both SWCNTs-P-F-A and SWCNTs-P-F-A-Nb were internalized in NNV-infected cells, and a stronger green fluorescence was observed in SWCNTs-P-F-A-Nb group. The internalization was quantitatively measured by flow cytometry analysis. As shown in Figure 3B, the percentage of positively labeling cells was 91.2% in SWCNTs-P-F-A-Nb group, while that was 65.5% for SWCNTs-P-F-A treatment. Previously, the internalization of MWCNTs (uncombined with Nb) in SSN-1 cells was checked, and results showed that the percentage of positively labeling cells was 32.57% (30). The data indicated that SWCNTs have a stronger penetrability than MWCNTs, which is consistent with a previous study (40). To further verify NNV targeting, the specific recognition and binding ability of SWCNTs-P-A-Nb to NNV was checked by transmission electron microscopy (TEM) observation. As shown in Figure 3C, the specific binding of virions with SWCNTs-P-A-Nb can be observed, indicating that the increased internalization of SWCNTs-P-A-Nb in SSN-1 cells is correlated with the specific binding of Nb to NNV.

**FIG 4:**
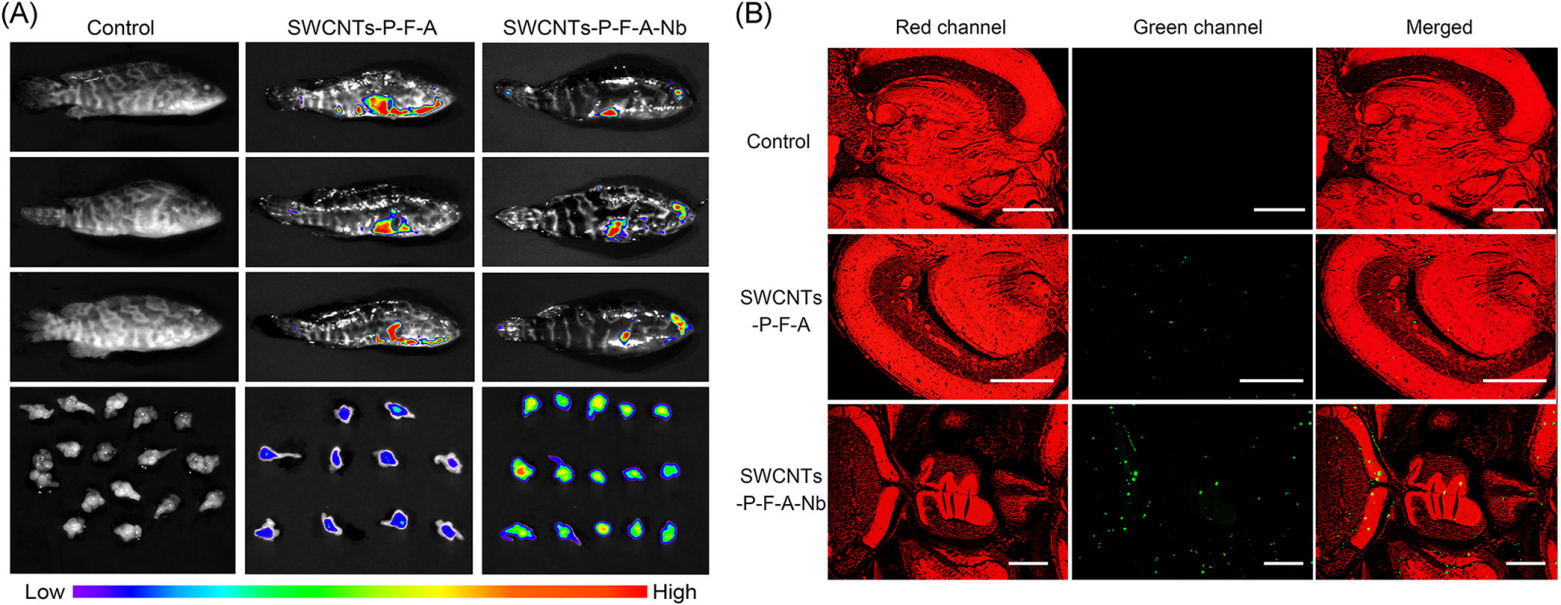
NNV targeting of the drug delivery system *in vivo*. (A) NNV-infected grouper juveniles treated with SWCNTs-P-F-A or SWCNTs-P-F-A-Nb with the same FITC concentration (5.0 μg/mL) by immersion and observed using an imaging system. Healthy grouper juveniles treated without SWCNTs-P-F-A or SWCNTs-P-F-A-Nb were used as the control. (B) Tissue section observation of the brain of the treated grouper. Scale bars: 500 μm.

For NNV targeting *in vivo*, SWCNTs-P-F-A was distributed mainly in the abdomen, while SWCNTs-P-F-A-Nb could enter into the head in addition to the abdomen (Fig. 4A). Moreover, the fluorescence in abdomen for SWCNTs-P-F-A-Nb groups was weaker than that for SWCNTs-P-F-A groups, indicating that the nonspecific distribution is reduced following NNV-Nb conjugation. The brains were collected and subjected to fluorescence imaging (Fig. 4A) and tissue section observation (Fig. 4B). Stronger fluorescent signals were observed following SWCNTs-P-F-A-Nb treatment than in the SWCNTs-P-F-A treatment group, indicating that more SWCNTs-P-F-A-Nb can enter into the brain tissue of diseased grouper. All the results indicated that SWCNTs-P-A-Nb has a good NNV-targeting ability *in vitro* and *in vivo* and improves the specific distribution in the NNV-infected sites under the guidance of NNV-Nb.

### Potential ways for ingestion and excretion.

**FIG 5:**
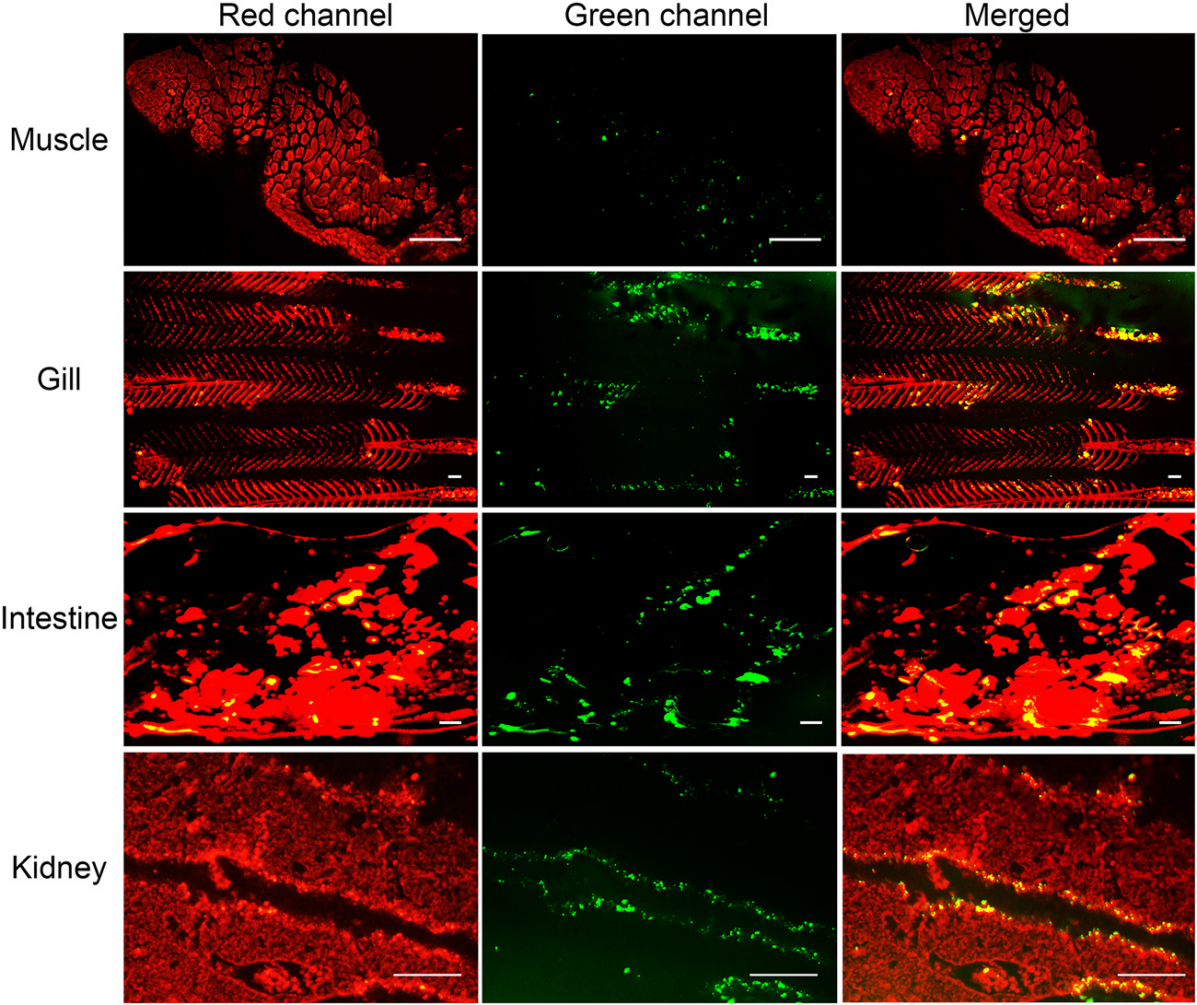
Potential ways for ingestion and excretion of SWCNTs-P-F-A-Nb. Scale bars: 500 μm.

As shown in Figure 5, SWCNTs-P-F-A-Nb was distributed in the muscle, gill, intestine, and kidney of the NNV-infected grouper. Distribution in muscle indicates that SWCNTs-P-F-A-Nb can effectively penetrate the epidermis and muscle by immersion. In addition, SWCNTs-P-F-A-Nb may also enter into the circulatory system by crossing the membranes of gill and intestine. Similar phenomena have been reported by previous studies. For example, Zhu et al. (35) checked the distribution of SWCNTs in rare minnow (Gobiocypris rarus) and found that SWCNTs can enter into the body through the skin and accumulate in the muscle (35). Moreover, another study found that nanoparticles can pass through the membranes of gill/intestine and enter into the circulatory system (41). Distribution in kidney indicates that SWCNTs-P-F-A-Nb can be excreted by kidney. Zhang et al. (23) constructed a targeted SWCNTs-based vaccine delivery system (SWCNTs-MG) for spring viremia of carp therapy. Their results showed that the SWCNTs-MG is distributed in the muscle, intestine, kidney, spleen, and liver of the diseased common carp, which is similar to the present results.

### Anti-NNV activity of the drug delivery system.

Targeted drug delivery platforms have been widely explored for disease therapy (17, 19). However, the most mature area lies in cancer therapy; applications against CNS viral diseases are rarely reported (23). To counter this, a nanobody-mediated NNV-targeting drug delivery platform is constructed to explore the application of targeted therapy in CNS viral diseases.

**FIG 6:**
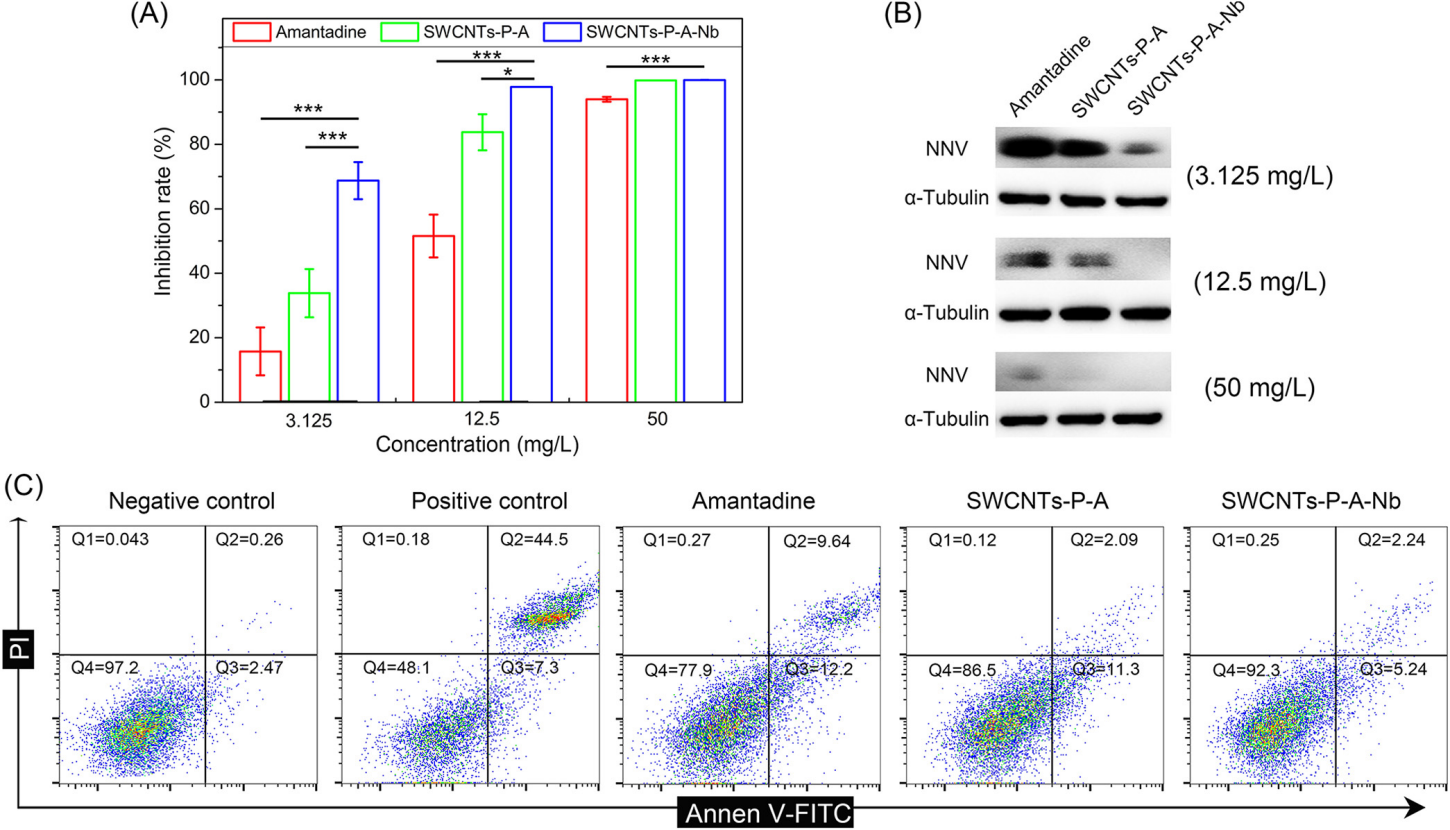
Anti-NNV activity of the drug delivery system *in vitro*. Anti-NNV activity of amantadine, SWCNTs-P-A, and SWCNTs-P-A-Nb analyzed by RT-qPCR (A) (*n *= 6 per group) and Western blotting (B). Data are presented as mean ± SD. *, *P ≤ *0.05 and ***, *P ≤ *0.001. (C) Apoptosis analysis of SSN-1 cells following different treatments (12.5 mg/L amantadine) by annexin V/PI staining. The annexin V-FITC^−^/PI^−^, annexin V-FITC^+^/PI^−^, and annexin V-FITC^+^/PI^+^ populations were regarded as normal cells, early apoptosis, and late apoptosis, respectively.

**FIG 7:**
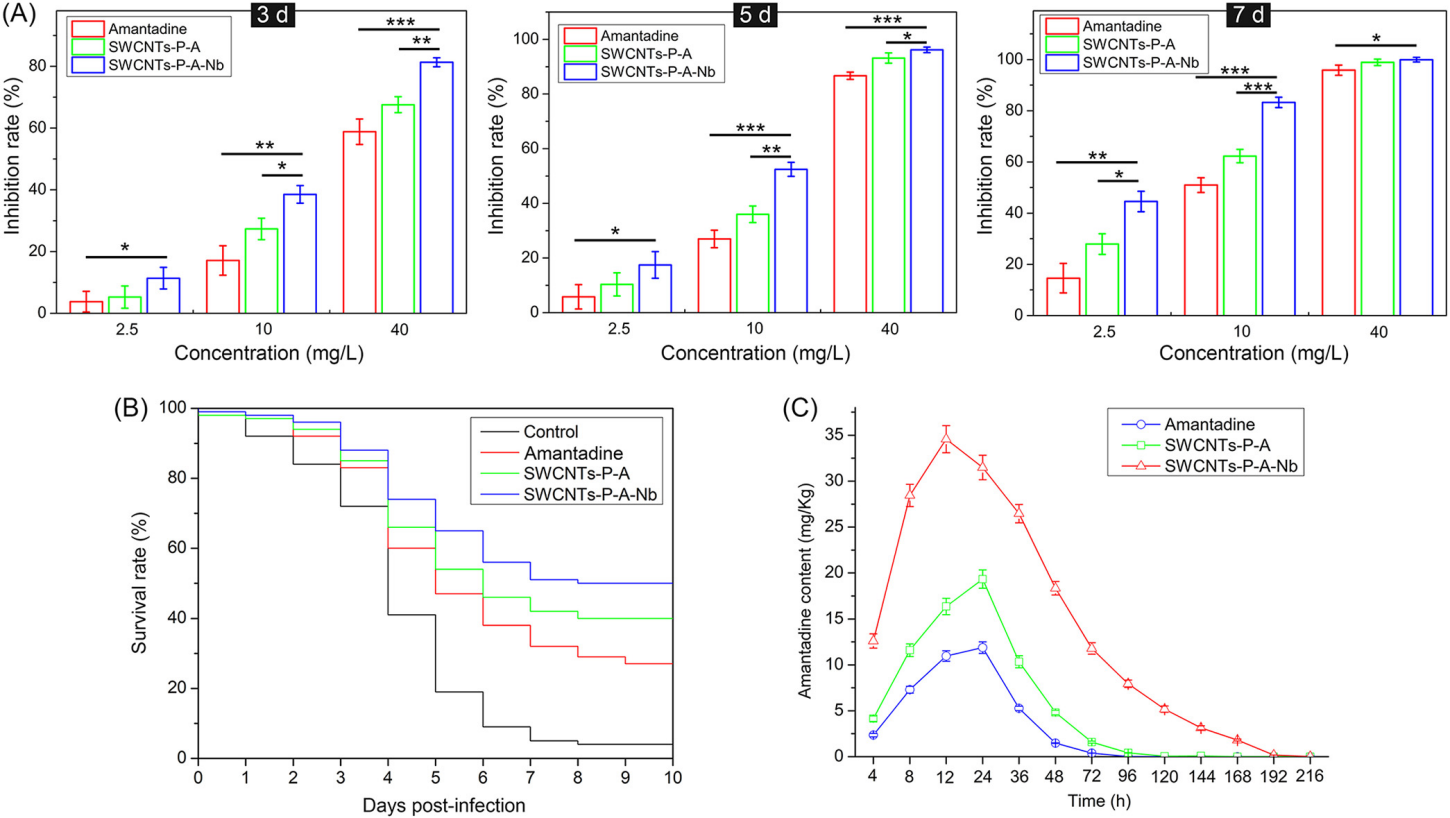
Anti-NNV activity of the drug delivery system *in vivo*. (A) Anti-NNV activity of amantadine, SWCNTs-P-A, and SWCNTs-P-A-Nb *in vivo*. *n *= 9 fish per group. (B) Survival curves of the infected grouper juveniles with different treatments (40 mg/L amantadine). The infected grouper juveniles without treatment were performed as the control. *n *= 100 fish per group. (C) Metabolism of amantadine in brain of grouper juveniles with different treatments (40 mg/L amantadine). *n *= 9 fish per group. Data are presented as mean ± SD. *, *P ≤ *0.05, **, *P ≤ *0.01, and ***, *P ≤ *0.001.

Toxicity of the drug delivery system was first evaluated, and data showed that both SWCNTs-P-A and SWCNTs-P-A-Nb were safe to SSN-1 cells and grouper juveniles under the concentrations used in the study. As shown in Figure 6A and B, SWCNTs-P-A-Nb showed an anti-NNV file stronger than that of amantadine alone or SWCNTs-P-A group. NNV infection induces host cell death, which generally indicates mitochondria-mediated caspase-dependent cell apoptosis (25). Therefore, apoptosis analysis was performed to check the protective effects on SSN-1 cell from NNV-induced apoptosis. As shown in Figure 6C, compared with those in the negative-control group (NNV-uninfected), the percentages of early (7.3%) and late apoptosis (44.5%) were significantly increased in the positive-control group (NNV-infected). The percentages of normal cells were 77.9%, 86.5%, and 92.3% for amantadine, SWCNTs-P-A, and SWCNTs-P-A-Nb treatments with the same amantadine concentration (12.5 mg/L), respectively. The data indicate that SWCNTs-P-A-Nb has the best protective effect on SSN-1 cells from NNV-induced apoptosis. Similarly, compared with amantadine and SWCNTs-P-A, SWCNTs-P-A-Nb also showed the best anti-NNV activity *in vivo* (Fig. 7A). As shown in Figure 7B, the survival rate of grouper juveniles was only 4% following infection with NNV for 10 days and increased, respectively, to 27%, 39%, and 51% after amantadine, SWCNTs-P-A, and SWCNTs-P-A-Nb treatments with the same amantadine concentration (40 mg/L).

Metabolism of amantadine in brain was analyzed using a liquid chromatography-mass spectrometer. As shown in Figure 7C, the maximum amantadine contents were reached at 24 h after exposure to amantadine (11.88 mg/kg) or SWCNTs-P-A (19.34 mg/kg), while that was reached at 12 h for SWCNTs-P-A-Nb treatment (34.57 mg/kg). After transferring to seawater without drug, the contents of amantadine were decreased. There was no detectable signal at around 120 h after exposure to amantadine or SWCNTs-P-A, while that lasted to 192 h for SWCNTs-P-A-Nb treatment. These data indicate that SWCNTs-P-A-Nb can transport amantadine to the brain in a fast and efficient manner and prolong the action time of amantadine. A similar result has been reported by previous studies (17, 42). For example, Ren et al. (42) have constructed a dual-targeting drug delivery system (O-MWCNTs-PEG-ANG) for brain glioma treatment. They demonstrated that the O-MWCNTs-PEG-ANG can greatly increase the accumulation of doxorubicin in brain (42). Targeted drug delivery system can transport and release drugs to the specific sites and decrease the distribution in normal tissues, thereby reducing toxicity and side effects and prolonging the circulation time, ultimately improving the effects of drug therapy (9, 10).

The above results show that SWCNTs-P-A-Nb has a stronger anti-NNV activity than amantadine or SWCNTs-P-A treatment group with the same amantadine concentration. Therefore, it can be concluded that SWCNTs-P-A-Nb can efficiently transport and release amantadine at the NNV-infected sites under the guidance of NNV-Nb, improving the anti-NNV activity of amantadine.

### Conclusions.

In summary, the potential application of targeted drug delivery in CNS viral disease therapy was explored using VER as a disease model. A nanobody-mediated virus-targeting drug delivery platform was constructed employing SWCNTs, amantadine, and NNV-Nb as the nanocarrier, anti-NNV drug, and targeting ligand. SWCNTs-P-A-Nb has a good NNV-targeting ability and can improve the anti-NNV activity of amantadine, showing a bright future for the CNS viral disease therapy.

## MATERIALS AND METHODS

### Cells, virus, and fish.

SSN-1 cells derived from the whole fry tissue of Channa striatus were obtained from Shenzhen Exit & Entry Inspection and Quarantine Bureau (Shenzhen, China) and maintained at 25°C in Lebovitz-15 medium (L-15; Gibco, USA) supplemented with 10% fetal bovine serum (FBS; ZETA LIFE, USA). NNV used in the study was previously isolated from diseased grouper, which belongs to the red-spotted grouper nervous necrosis virus genotype (30). Healthy hybrid grouper (Epinephelus fuscoguttatus♀ × Epinephelus lanceolatus♂) juveniles (5 to 6 weeks) were obtained from Maoming Binhai New Area Chenxi Biotechnology Co., Ltd. (Maoming, China). All animal experiments in the study were approved by the Animal Ethical and Welfare Committee of Northwest A&F University (Yangling, China).

### Materials.

Pristine SWCNTs (P-SWCNTs) were purchased from Chengdu Organic Chemicals Co., Ltd., Chinese Academy of Sciences (Chengdu, China). Amantadine, polyethylenimine (PEI), fluorescein isothiocyanate (FITC), and trypan blue were purchased from Shanghai Aladdin Biochemical Technology Co., Ltd. (Shanghai, China). Dimethyl sulfoxide (DMSO), N-hydroxysuccinimide (NHS), 1-ethyl-3-(3-dimethylaminopropyl) carbodiimide hydrochloride (EDAC), 2-(N-morpholino) ethanesulfonic acid (MES), N,N-dimethylformamide (DMF), and N,N-diisopropylcarbodiimide (DIC) were obtained from Sinopharm Chemical Reagent Co., Ltd. with a purity of ≥99%.

### Expression and affinity analysis of NNV-Nb.

Expression, purification, and affinity analysis of NNV-Nb were carried out according to the previous study (30). Briefly, the coding gene of NNV-Nb was cloned and ligated into pET-25b (+) and then transformed into Escherichia coli Rosetta (DE3) cells. The transformant was cultured in LB medium with 50 μg/mL carbenicillin at 37°C until the optical density at 600 nm (OD_600_) reached approximately 0.6. Subsequently, IPTG was added into the culture to a final concentration of 0.2 mM, and the transformant continued to be induced at 30°C for 6 h. Expression of NNV-Nb was analyzed by SDS-PAGE. NNV-Nb was purified with Ni-chelating affinity chromatography and analyzed by SDS-PAGE. Affinity activity of the purified NNV-Nb to NNV was checked by indirect ELISA. Briefly, 96-well plates were, respectively, coated with phosphate-buffered saline (PBS; as the blank control), bovine serum albumin (BSA; as the negative control), and NNV at 4°C overnight and then blocked with 3% BSA solution at 37°C for 1 h. A series of concentrations of NNV-Nb were added and incubated at 37°C for 1 h and then incubated with horseradish peroxidase (HRP)-conjugated rabbit anti-6×His tag antibody. TMB (3,3′,5,5′-tetramethylbenzidine) substrate (100 μL/well) was added and incubated at 37°C for 10 to 30 min, and the absorbance at 450 nm (OD_450_) was measured after the reaction was terminated by adding 2 M H_2_SO_4_. The results were identified as positive when the conditions of the following equation were met: (A_NNV_ − A_BC_)/(A_NC_ − A_BC_) >2, where A_BC_, A_NC_, and A_NNV_, respectively, refer to the OD_450_ of the blank control, negative control, and NNV-treatment group.

### Synthesis of constructs.

SWCNTs, amantadine, and NNV-Nb were selected to construct the targeted drug delivery platform. In brief, P-SWCNTs were oxidized by a H_2_SO_4_/HNO_3_ mixture (3:1, vol/vol) to form carboxyl groups on the surface of SWCNTs (O-SWCNTs). O-SWCNTs were modified with PEI (SWCNTs-PEI) to acquire well biocompatibility and water dispersibility and, importantly, provide a large amount of active amino groups for subsequent reactions (37). SWCNTs-PEI was conjugated with amantadine to obtain SWCNTs-P-A, and then that was conjugated with NNV-Nb to acquire SWCNTs-P-A-Nb. Finally, SWCNTs-P-A and SWCNTs-P-A-Nb were modified with FITC to acquire SWCNTs-P-F-A and SWCNTs-P-F-A-Nb, respectively. The loading efficiencies (percentage) of PEI, amantadine, Nb, and FITC were measured according to the previous study (30).

### Characterization of the constructs.

Morphological characteristics of SWCNTs-P-A-Nb were observed under a high-resolution TEM (HR-TEM; Tecnai G_2_ F20, USA). Elemental compositions of P-SWCNTs, O-SWCNTs, SWCNTs-PEI, and SWCNTs-P-A-Nb were analyzed by an X-ray photoelectron spectroscopy (XPS; PHI-5600, Russia). Thermogravimetric analysis (TGA) was performed to further qualitatively or quantitatively characterize the modification of SWCNTs. Particle sizes (nm) and zeta potentials (mV) of P-SWCNTs, O-SWCNTs, SWCNTs-PEI, and SWCNTs-P-A-Nb were measured by a Nano-particle size and Zeta potential analyzer (ZEN3600, Malvern, UK).

### NNV targeting *in vitro*.

SSN-1 cells were grown to a monolayer on coverslips in 12-well plates. Following infection with 10^2^ 50% tissue culture infective dose (TCID_50_) NNV for 24 h, the cells were exposed to FITC, SWCNTs-P-F-A, or SWCNTs-P-F-A-Nb with the same FITC concentration (1.0 μg/mL). After exposure for 4 h, the cells were washed with PBS and fixed in 4.0% paraformaldehyde. Cell membrane and nucleus were, respectively, dyed with Dil (5.0 mg/mL; Beyotime, China) and DAPI (4′,6-diamidino-2-phenylindole; 1.0 mg/L; Beyotime, China). After washing, the cells were observed and photographed under a confocal microscopy (Nikon, Japan).

For flow cytometry analysis, SSN-1 cells were grown to a monolayer in 6-well plates. Same treatments were performed as described above without staining with Dil or DAPI. Cells were thoroughly washed and analyzed using a BD FACSAria III flow cytometer (BD, USA). For TEM observation, SSN-1 cells were grown to a monolayer in culture flasks and infected with 10^2^ TCID_50_ NNV for 24 h. After exposure to SWCNTs-P-A-Nb for 4 h, the cells were collected and thoroughly washed. NNV targeting of SWCNTs-P-A-Nb was checked under a TEM (43) (JEOL, Tokyo, Japan).

### NNV targeting *in vivo*.

Following infection with 10^5^ TCID_50_ NNV for 3 days, grouper juveniles were treated with SWCNTs-P-F-A and SWCNTs-P-F-A-Nb with the same FITC concentration (5.0 μg/mL) by immersion. Based on the preexperiment, we found that SWCNTs-P-F-A-Nb can significantly enter the grouper and stably distribute in various tissues following immersion for 12 h. Therefore, the grouper juveniles were thoroughly washed and observed using a multimode *in vivo* imaging system (AniView100, BioLight, Guangzhou, China) following treatment for 12 h. In addition, brains of the grouper juveniles were collected and subjected to fluorescence imaging and tissue section observation using a fluorescence stereomicroscope (Leica MZFL III, Germany) (30). Healthy grouper juveniles treated without SWCNTs-P-F-A or SWCNTs-P-F-A-Nb were used as the controls.

### Ingestion and excretion analysis.

Following treatments as described in the section above, the grouper juveniles were killed. The muscle, gill, intestine, and kidney were collected for tissue section observation using the fluorescence stereomicroscope to check the potential ways for ingestion and excretion of SWCNTs-P-F-A-Nb.

### Anti-NNV activity of the drug delivery system *in vitro*.

To evaluate the anti-NNV activity of the drug delivery system *in vitro*, toxicity of SWCNTs-P-A and SWCNTs-P-A-Nb to SSN-1 cells was first checked by trypan blue staining test (44). SSN-1 cells were grown to a monolayer in 12-well plates and infected with 10^2^ TCID_50_ NNV. Following infection for 2 h, the cells were, respectively, treated with amantadine, SWCNTs-P-A, and SWCNTs-P-A-Nb with the same amantadine concentrations (3.125, 12.5, and 50 mg/L). Following treatment for 48 h, the relative NNV contents in SSN-1 cells were analyzed by reverse transcriptase quantitative PCR (RT-qPCR) and Western blotting. Moreover, apoptosis analysis was carried out based on Annexin V/propidium iodide (PI) staining (Beyotime Biotech, Nantong, China). Briefly, SSN-1 cells were grown to a monolayer in 6-well plates and treated as described above. Approximately 2 × 10^5^ cells were collected and stained with annexin V-FITC (5 μL) and PI (5 μL) following the manufacturer’s instructions. After staining, flow cytometry analysis was conducted immediately. FITC fluorescence (FL1) and PI fluorescence (FL2) of each cell were quantitated using the Cell Quest Pro software. The annexin V-FITC^−^/PI^−^, annexin V-FITC^+^/PI^−^, and annexin V-FITC^+^/PI^+^ populations were regarded as normal cells, early apoptosis, and late apoptosis, respectively.

### Anti-NNV activity of the drug delivery system *in vivo*.

Toxicity of SWCNTs-P-A and SWCNTs-P-A-Nb to grouper juveniles was first evaluated. For anti-NNV activity evaluation, healthy grouper juveniles were infected with 10^5^ TCID_50_ NNV by intraperitoneal injection for 1 day and then, respectively, treated with amantadine, SWCNTs-P-A, and SWCNTs-P-A-Nb with the same amantadine concentrations (2.5, 10, and 40 mg/L) by immersion. Grouper juveniles infected with NNV and treated without drugs were used as the control. Nine grouper juveniles were randomly collected at 3, 5, and 7 days postinoculation (dpi), and the brain was collected for NNV contents analysis. The same treatment was performed to evaluate the protective effects of amantadine, SWCNTs-P-A, and SWCNTs-P-A-Nb on grouper from NNV infection over a 10-day period.

### Amantadine metabolism.

Grouper juveniles were infected with 10^5^ TCID_50_ NNV by intraperitoneal injection for 3 days and then, respectively, treated with amantadine, SWCNTs-P-A, and SWCNTs-P-A-Nb with the same amantadine concentration (20 mg/L) by immersion. Following exposure for 24 h, the grouper juveniles were transferred to clean seawater without drug. The brains were collected at the specific time points and fully ground by a homogenizer. After centrifugation for 30 min, the supernatants were collected. Contents of amantadine in the supernatants were measured using a liquid chromatography-mass spectrometer (Thermo Scientific, USA) according to a previous study (45).

### Statistical analysis.

Data were presented as mean ± standard deviation (SD). Statistical comparisons between two groups were performed using the two-tailed unpaired Student’s *t* test, and comparison between more than two groups was analyzed with one-way analysis of variance (ANOVA). Values of *, *P ≤ *0.05, **, *P ≤ *0.01, and ***, *P ≤ *0.001 were applied to annotate statistical significance.
